# Ageing-associated small RNA cargo of extracellular vesicles

**DOI:** 10.1080/15476286.2023.2234713

**Published:** 2023-07-27

**Authors:** Fabian Kern, Thomas Kuhn, Nicole Ludwig, Martin Simon, Laura Gröger, Natalie Fabis, Ernesto Aparicio-Puerta, Abdulrahman Salhab, Tobias Fehlmann, Oliver Hahn, Annika Engel, Viktoria Wagner, Marcus Koch, Katarzyna Winek, Hermona Soreq, Irina Nazarenko, Gregor Fuhrmann, Tony Wyss-Coray, Eckart Meese, Verena Keller, Matthias W. Laschke, Andreas Keller

**Affiliations:** a Chair for Clinical Bioinformatics, Saarland Informatics Campus, Saarland University, Saarbrücken, Germany; b Helmholtz-Institute for Pharmaceutical Research Saarland (HIPS), Helmholtz-Centre for Infection Research (HZI), Department for Clinical Bioinformatics, Saarbrücken, Germany; c Helmholtz-Institute for Pharmaceutical Research Saarland (HIPS), Helmholtz-Centre for Infection Research (HZI), Biogenic Nanotherapeutics Group (BION), Saarbrücken, Germany; d Department of Pharmacy, Saarland University, Saarbrücken, Germany; e Department of Human Genetics, Saarland University, Homburg, Germany; f Center for Human and Molecular Biology, Saarland University, Homburg, Germany; g Molecular Cell Biology and Microbiology, Wuppertal University, Wuppertal, Germany; h Department of Genetics and Epigenetics, Saarland University, Saarbrücken, Germany; i Department of Neurology and Neurological Sciences, Stanford University, Stanford, USA; j INM – Leibniz Institute for New Materials, Saarbrücken, Germany; k The Edmond and Lily Safra Center for Brain Sciences, The Hebrew University of Jerusalem, Jerusalem, Israel; l Faculty of Medicine, Institute for Infection Prevention and Control; Medical Center - University of Freiburg, Freiburg, Germany; m Institute for Clinical and Experimental Surgery, Saarland University, Homburg, Germany; n Center for Bioinformatics, Saarland University, Saarbrücken, Germany

**Keywords:** Noncoding RNA, tissue ageing, extracellular vesicles, plasma, miRNA

## Abstract

Previous work on murine models and humans demonstrated global as well as tissue-specific molecular ageing trajectories of RNAs. Extracellular vesicles (EVs) are membrane vesicles mediating the horizontal transfer of genetic information between different tissues. We sequenced small regulatory RNAs (sncRNAs) in two mouse plasma fractions at five time points across the lifespan from 2–18 months: (1) sncRNAs that are free-circulating (fc-RNA) and (2) sncRNAs bound outside or inside EVs (EV-RNA). Different sncRNA classes exhibit unique ageing patterns that vary between the fcRNA and EV-RNA fractions. While tRNAs showed the highest correlation with ageing in both fractions, rRNAs exhibited inverse correlation trajectories between the EV- and fc-fractions. For miRNAs, the EV-RNA fraction was exceptionally strongly associated with ageing, especially the miR-29 family in adipose tissues. Sequencing of sncRNAs and coding genes in fat tissue of an independent cohort of aged mice up to 27 months highlighted the pivotal role of miR-29a-3p and miR-29b-3p in ageing-related gene regulation that we validated in a third cohort by RT-qPCR.

## Introduction

Understanding and controlling the molecular hallmarks of age-related processes in higher organisms promises to greatly improve the quality of life [1]. For humans, ageing is frequently studied using easily accessible biospecimens such as blood, serum, or urine. Consequently, the scientific community generated models for a broad spectrum of molecular physiological and pathophysiological processes from different molecular types. For example, studies rely on long-lived individuals [2], serum proteomic profiling [3], small RNA patterns in blood cells [4,5], or the exploration of epigenetic control of ageing clocks [6]. Likewise, deeper profiles, such as gene expression fingerprints, are available for different tissues [7]. Murine models facilitate the analysis of such processes thanks to their restricted influence of genetic background and varying lifestyles compared to humans. In mouse models, the aged immune system drives senescence and ageing of solid organs [8]. Further, organism-wide RNA-sequencing data of major organs and cell types across the mouse lifespan provide an important resource to study ageing [9,10]. The available data suggest complex ageing patterns, including both linear and non-linear effects that are either specific for organs or follow global organism-wide trajectories. Ageing and parabiosis-mediated rejuvenation suggest an almost universal loss of gene expression with age that is largely mimicked by heterochronic parabiosis: aged blood reduces global gene expression, and young blood restores it in select cell types [11]. In the same direction, Sahu and co-workers demonstrated that a beneficial effect of young blood on aged muscle regeneration was diminished when serum was depleted of extracellular vesicles (EVs), indicating the important role of EVs in ageing and rejuvenation [12]. In addition to blood, also young CSF has a beneficial effect by restoring oligodendrogenesis and memory in aged mice [13].

These observations indicate a systemic and orchestrated exchange of information and molecules between organs. For example, extracellular vesicles (EVs) are membrane vesicles mediating the horizontal transfer of genetic information between different cell types. Specifically, EVs are postulated to play an important role [14,15] in, e.g. hypothalamic stem cells seem to control ageing through EV miRNAs [16]. Recently, targeted intervention of EV-mediated transfer of miRNAs from osteoclasts to chondrocytes was described as a promising method to slow or even inhibit osteoarthritis in mice [17]. Furthermore, several studies have addressed the relationship of EVs with ageing in a systematic manner [18–21]. Complementary studies have investigated the change in EV-bound noncoding RNAs depending on (treatment) interventions such as caloric restriction [22]. However, these are limited in their analysis scope by considering only one small RNA class at a time, often even to a subset of well-characterized representatives. Moreover, an inherent restriction is the limited sample volume, frequently leading to pooling of biosamples and blurring of fine-grained signals.

These and other issues complicate the analysis of EVs and their molecular cargos. Especially in the context of EVs in cancer, common pitfalls in purification have been summarized by Schekman and co-workers [23], with the correct nomenclature of EVs, purification and other aspects elucidated in great detail. Considering these inconsistencies, we use the term ‘extracellular vesicles’ (EVs) throughout the manuscript as recommended by the International Society for EVs. EV means the full fraction of vesicles up to 400 nm in diameter irrespective of their origin and biogenesis.

**Figure 1.**
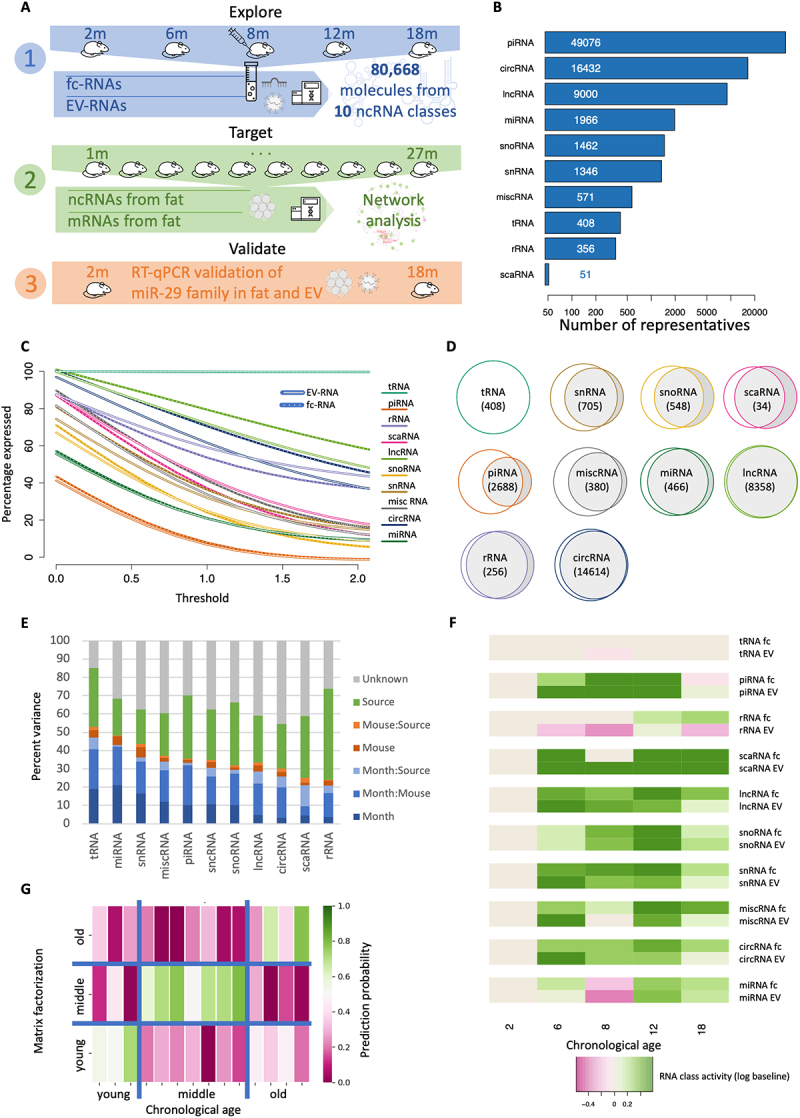
Distribution of sequencing reads into their mapped noncoding RNA classes and their relation to ageing across the mouse lifespan. (a) Study setup. We profiled EV- and fc fraction vesicle and plasma samples from mice in five age groups, sequenced 80,668 noncoding RNAs from 10 classes (1), sequenced fat tissue specimens from TMS as an independent cohort (2) and validated the key findings using RT-Qpcr in another independent cohort (3). (b) Overall distribution of molecules to the 10 noncoding RNA classes under investigation. (c) Fraction of representatives per RNA class (y-axis) exceeding the expression threshold (x-axis; normalized counts). The RNA class is presented as solid line in the foreground. Dashed with lines indicate the fc fraction and solid lines the EV fraction. (d) Overlap of expressed RNAs in EVs and in the fc fraction as area proportional Venn diagrams. (e) Percent of total data variance attributed to different parameters, such as age (month), individual mice or specimen type (source). Columns are sorted according to decreasing fraction of variance attributed to age. (f) Relative expression of the different RNA classes per time point and sample type compared to the baseline (2 months). Green indicates higher expression, and purple indicates lower expression. The upper row per RNA class shows the fc fraction, and the lower row shows the EV fraction. (g) Prediction of age by nonnegative matrix factorization. The colour code represents the probability (trust) in the prediction, the x-axis represents the true age group, and the y-axis represents the predictions.

The main aim of our study was to provide a data resource of small non-coding RNAs included in EV cargo and freely circulating in plasma (fc-RNAs) in mice of different ages and to identify differences between the molecular information in these fractions associated with ageing that might advance our understanding of the systemic ageing process. Therefore, plasma fc-RNA and EV-RNA of individual mice were sequenced for noncoding RNA profiling and contrasted by computational approaches. To demonstrate the use of this resource, we conducted pathway and comparative analyses using original Tabula Muris senis (*TMS)* data [9,10] and performed sequencing of small RNAs from TMS as an independent cohort. Finally, we validated the core findings by RT-qPCR in a third cohort of aged mice (Figure 1a).

## Results

### Noncoding RNAs are modulated specifically upon ageing in EV- and fc-RNA samples

To uncover age-related dynamic processes and to model the information exchange involved, we sequenced both non-coding fc-RNAs and non-coding EV-RNAs from individual mice. The molecular profiles are available at five time points across the average lifespan between two and 18 months in two to four replicates per age group and biospecimen type (Supplementary Table S1). For the fc- and EV-RNA samples, we sequenced an average of 38 million reads per sample and mapped them to ten different noncoding RNA classes. Our analysis covered a total of 80,688 different noncoding RNAs, with piRNAs, circRNAs, lincRNAs and miRNAs being the classes with the highest number of different features (Figure 1b). The first aspect of the analysis encompassed the distribution of molecules from the different RNA classes. While tRNA fragments were highly represented both in EV- and fc-fractions, piRNAs showed sharply lower levels in both specimen types (Figure 1c). However, varying amounts of circRNAs and rRNAs were predominantly observed in the EV- and fc-fractions. Notably, this analysis has a quantitative and RNA class-centric view but does not yet consider whether the representatives within the classes match across sample types. For example, only a small number of piRNAs were present in both the EV and fc fractions, even though the general abundance was high. Considering the sample type overlap for each class, the most significant difference was indeed observed between fc- and EV-bound piRNAs (Figure 1d). Similarly, we report large differences in the content of RNA molecules from snRNAs, snoRNAs, and scaRNAs. In contrast, detected tRNA fragments, lincRNAs, rRNAs and circRNAs are often shared between the two fractions. In summary, our data argue for type-specific expression patterns that differ significantly between noncoding RNA classes both in a quantitative and qualitative manner.

We thus asked whether unique ageing trajectories within and between noncoding RNA classes exist, either enclosed into/bound to EVs or freely circulating in plasma. One indicator is the proportion of variance in the RNA counts that can be explained by available sample covariates, i.e. either by age of the mice, the specimen type and donor mice identity, or linear combinations of such. Depending on the RNA class we observe varying results with respect to the separation in the fc- and EV-fraction in a 2-dimensional UMAP embedding, with a clear segregation in the scaRNAs (Supplementary Figure S1). Compared to the other RNA classes, tRNA fragments and miRNAs however showed the highest fraction of variance explained by age (Figure 1e). In comparison, the lowest variation with respect to age was observed for scaRNAs and rRNAs. Importantly, the individuality factor of each donor mouse used for this study was comparably small and independent of the RNA class. To uncover a potential relationship between each RNA class and mouse age, we used the expression at month 2 as baseline and modelled whether it increases or decreases over time for EV-RNA and fc-RNA separately but observed similar dynamics of change.

The largest age-related differences appear for rRNAs, where the overall amount increases for free circulating molecules with ageing but the EV loading of rRNAs decreases. This notable difference in the specimen types also explains the high proportion of variance attributed to the sample type annotation (Supplementary Figure S2). Our data further indicate a strong ageing signal in EV-and fc fractions, with varying strengths, again depending on the RNA class (Figure 1f). As our previous analyses emphasized the role of tRNA fragments, we investigated the expression profiles in an unbiased manner and performed a classification into three age groups (young, 2 months; middle aged, 6–8 months; old, 12–18 months). We modelled this classification task as an optimization problem through nonnegative matrix factorization, computing probabilities for each sample to belong to each of the three groups. We then assigned each sample to the age group with the highest probability. For both fc- and EVs, we computed varying prediction accuracies, once more with the best results obtained for tRNA fragments with a remarkable accuracy of 86% (Figure 1g).

Taken together, our analyses suggest that non-coding RNAs exhibit specific age trajectories, both qualitatively and quantitatively. Moreover, the data pinpointed substantial differences in the case of fc-RNAs and EV-RNAs, where the highest correlation with age was observed for tRNA fragments. This poses the question of whether loading of EVs follows biologically relevant environmental mechanisms. To potentially discover such patterns, we next performed a fine-granular and molecule-centric analysis.

### Ageing patterns deviate within specific RNA classes

**Figure 2.**
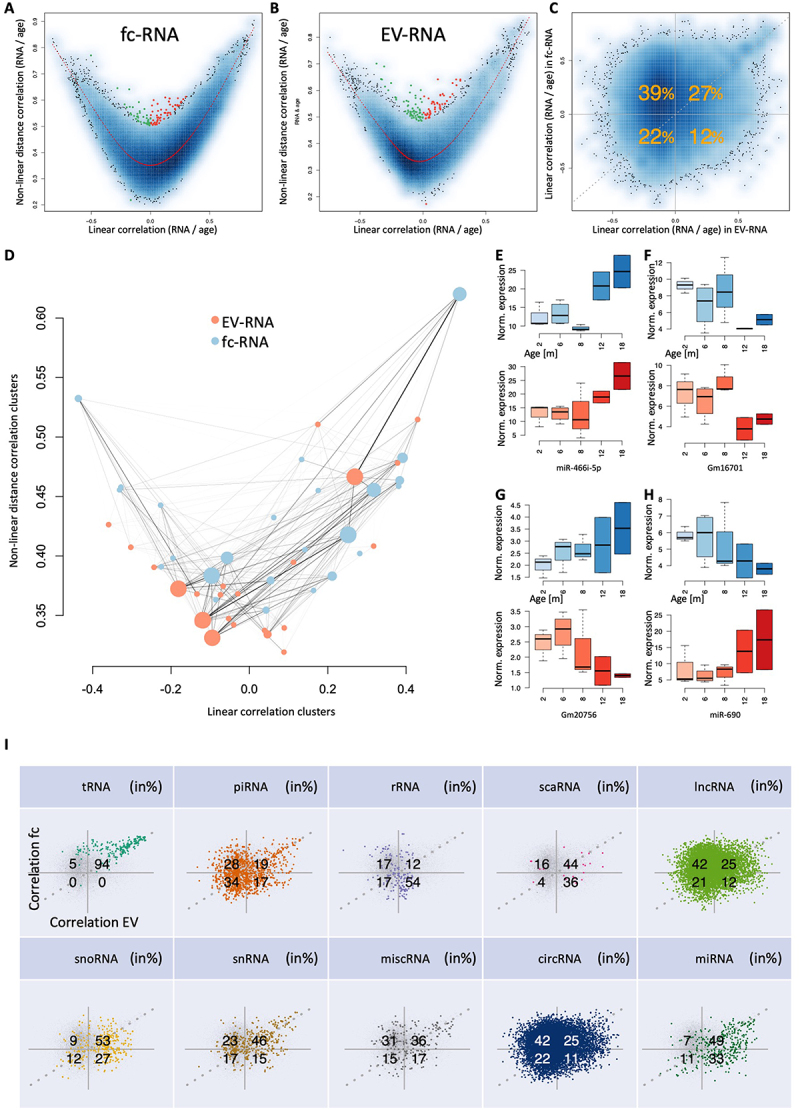
Correlation of noncoding RNAs with ageing in fc- and EV fractions. (a) for each noncoding RNA, the x-axis represents the Pearson correlation, and the y-axis represents the distance correlation with age in the fc fraction. The colour gradient in the background represents the density of non-coding RNA representatives with respective linear and non-linear correlation. The red line is a smoothed spline. The coloured dots (green, negatively; red, positively) are correlated with age in a predominantly nonlinear manner, i.e. those points are with a distance of at least 0.15 away from the spline. (b) the same information as in panel (a) but for EVs. (c) Scatter plot showing the Pearson correlation in EVs (x-axis) in relation to the Pearson correlation in the fc fraction (y-axis). Orange numbers represent the percentage of points in each of the four quarters. The data suggest a shift to a negative correlation with age in EVs. Numbers are rounded to integers. (d) Noncoding RNAs in the fc- and EV fractions were clustered, and the resulting clusters were attributed to the average linear and nonlinear correlation. Solid lines represent a match of noncoding RNAs; the thicker the line is, the more noncoding RNAs match between an fc and EV clusters. The diameter of the points represents the cluster size. Most EV clusters accumulate in the lower left corner. (e-h) Examples of noncoding RNAs that increase or decrease with age. The x-axis represents the age, and the y-axis represents the expression of the selected noncoding RNA (orange, EV fraction; blue, fc). Colored boxes span the first to the third quartile, with the line inside the box representing the median value. The whiskers show the minimum and maximum values or values up to 1.5 times the interquartile range below or above the first or third quartile if outliers are present. (i) Confusion matrix scatter plots (see also panel (c)) split by the RNA classes.

Of the 80,668 unique noncoding RNA molecules in *Mus musculus* included in our analysis, 23052 (28.6%) were stably present in the EV- and fc-fractions (Supplementary Table S2). We then computed the linear Pearson correlation as well as the nonlinear distance correlation for each of the investigated RNAs. While making conclusions on the correlations of single RNA features can be challenging in terms of type-I errors, comparing the different RNA classes and the fc- and EV-fractions globally can support an understanding of linear and non-linear ageing effects. We thus computed an estimate for each RNA whether it was linearly correlated with age, nonlinearly correlated with age, or not correlated with age at all for EV-and fc-fractions separately. Because of the large number of non-coding RNA features included in the study we are potentially facing an overplotting issue and thus computed a density estimation for the two correlation schemes. For both sample types, fc- (Figure 2a) and EV- (Figure 2b), the linear component was dominant, and only a few exceptions with nonlinear trajectories occurred. Those are characterized by a distance of at least 0.15 from a spline with eight degrees of freedom. The amplitude and frequency of nonlinear RNAs were both slightly enriched in EVs. Interestingly, we also observed a pattern towards a slightly negative correlation in EVs as compared to a positive correlation (average of 0.106) in the fc fraction. To test whether the average of 0.106 is the result of a random effect we performed permutation tests. Here, we reached an average correlation of −0.0003, marking a statistically significant difference (*p* < 10^−16^). Of note, most of the correlation values observed in our study are not significant. Generally, linear correlation coefficients above 0.5 and −0.5 in our study roughly corresponded to p-values with nominal significance at an alpha level of 0.05 (Supplementary Table S2).

Having observed noncoding RNAs that are either positively and negatively correlated with age in EV and fc fractions further called for exploring whether the up- and downregulated candidates show similar compositions in the two specimen types. In total, 27% and 22% increased and decreased with age in the EV fraction and fc fraction, respectively, slightly differing from what would be expected by a random distribution. However, 39% of the 23,052 expressed noncoding RNAs were negatively correlated with age in EVs but positively correlated in the fc fraction while only 12% presented the opposite behaviour, i.e. were negatively correlated with age in the fc- fraction and positively correlated with age in the EV-fraction (Figure 2c, Supplementary Table S2). To seek common patterns for the increasing and decreasing expression of non-coding RNAs, we clustered the expression in EV-and fc-fractions separately and extracted RNA clusters from the dendrogram. For each cluster, we then computed the average linear and nonlinear correlation with ageing and finally calculated the overlap of the sample types. Our analysis confirmed a strong decrease in the correlation with age in the EV fraction compared to the fc fraction (Figure 2d). The EV clusters are enriched in the lower left corner, indicating a significant trend towards a negative correlation with age in EVs. Furthermore, the data reveal an age-related loss in linear correlation compared to non-linear correlation. To validate the origin of these signals, we inspected all concordant and discordant noncoding RNAs and provide specific examples for markers clearly increasing and decreasing with age in both EV and fc fractions *(miR-466i-5p*, Figure 2e *and Gm16701*, Figure 2f, *respectively)*, decreasing with age in the EV fraction but increasing in the fc fraction *(Gm20756*, Figure 2g), and finally increasing with age in the EV fraction but decreasing in the fc fraction *(miR-690*, Figure 2h). We further examined whether the patterns hold for all 10 noncoding RNA classes or if they are rather class specific. Here, the specificity of patterns for the different non-coding RNA classes was astonishing. For example, 94% of tRNA fragments increased with age in both the EV and fc fractions. Additionally, 54% of rRNAs decreased with age in the fc fraction but increased with age if EV-bound. Conversely, 42% of circRNAs increased with age in the fc fraction but decreased with age if EV-bound (in- or outside). Additionally, other RNA classes revealed distribution patterns significantly deviating from the 25% per group as expected by chance. Finally, 82% of miRNAs increased with age in the EV fraction (Figure 2i).

### The miR-29 family controls ageing-related processes in fat tissues

**Figure 3.**
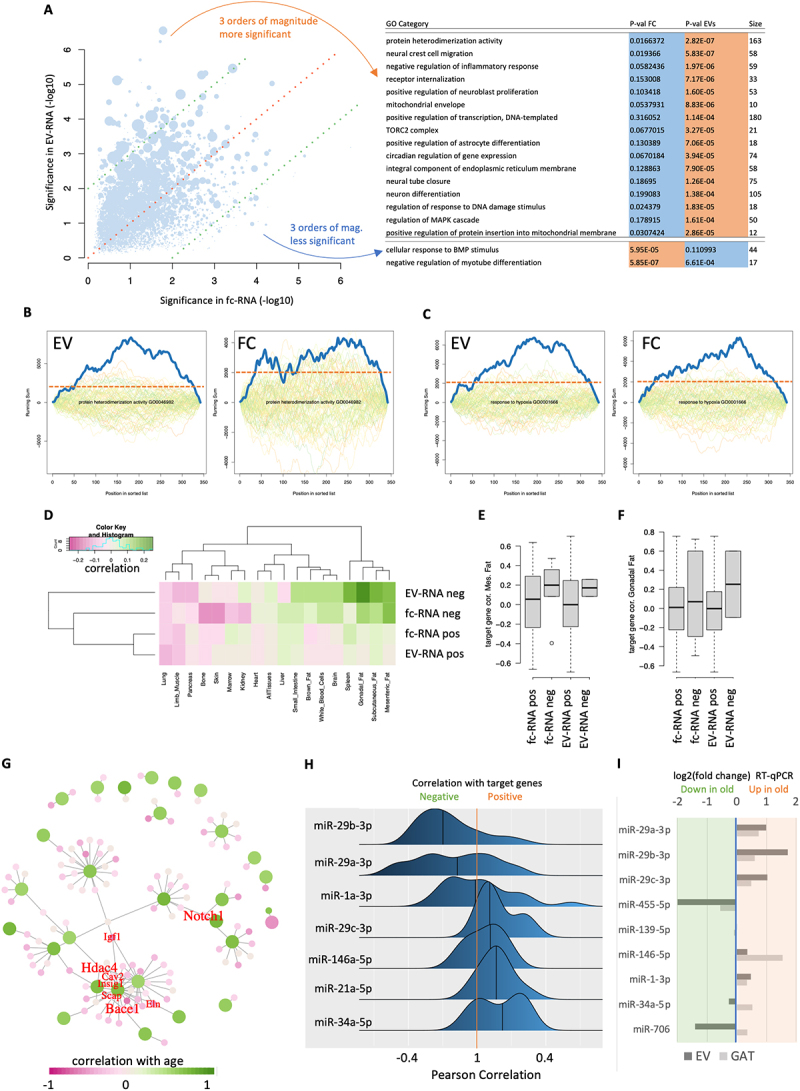
Pathway results and miRNA-target regulation networks. (a) miRNA pathway enrichment for age-related miRnas in the fc fraction (x-axis) and EVs (y-axis). Each dot is one pathway, and the size represents the number of miRnas associated with the pathway. The red dashed line is the bisector, and the green lines indicate two orders of magnitude higher significance in the fc fraction and in EVs. The pathways with at least a three orders of magnitude difference are listed on the right. (b) Enrichment plots for the category protein heterodimerization activity in the EV and fc fraction. The solid blue line denotes the enrichment for the miRnas sorted with respect to the correlation with age. The coloured lines in the background denote the enrichment plots for random distributions. The more the blue line exceeds the background distribution the more significant the pathway is enriched. The horizontal dashed orange line represents a running sum of 2,000 to make the two curves better comparable to each other. (c) Same information as in panel (b) for the response to hypoxia. Here, both, the Ev and fc fraction exceed the random distributions significantly. (d) for miRnas that are positively and negatively correlated with age in either the fc fraction or EVs, the average correlation with age of target genes from Tabula muris senis across 17 tissues is shown. As expected, miRnas decreasing with age showed target genes increasing with age and vice versa. (e, f) for two fat tissues, the target gene correlation with age is detailed for the four groups shown as rows in (b). Gray boxes span the first to the third quartile, with the line inside the box representing the median value. The whiskers show the minimum and maximum values or values up to 1.5 times the interquartile range below or above the first or third quartile if outliers are present (shown as separate, black outlined dots). (g) Target network. Large green dots depict miRnas, small pink dots represent genes, and lines delineate experimentally validated regulatory events between miRnas and genes. The colour shading represents the correlation with age, and the hub genes targeted by at least three miRnas are annotated in red. Relative font sizes represent the number of miRnas targeting the respective gene. (h) for the core miRnas from panel (g), the direct correlation of miRnas to all target genes in fat tissues of the TMS cohort is shown. Distributions are sorted with respect to an increasing average correlation such that the most consistent downregulation of target genes is observed for miRnas at the top. (i) RT-Qpcr results. The log2 fold-change based on the RT-Qpcr is presented for EV and GAT tissue. The miR-29 family members present a consistent up-regulation in both fractions.

In light of the regulatory role of miRNAs typically repressing gene expression [24] and further knowing that mRNA levels tend to decrease with age, we chose this particular class of noncoding RNAs to reveal further potential of our data resource. We first asked whether the miRNAs increasing or decreasing with age in EV- and fc-fractions exhibit distinct functions in a pathway-specific manner. For each gene ontology category [25], we computed an enrichment score for the miRNAs in EVs and in the fc fraction. In more detail, we used the list of miRNAs sorted by their correlation with age to perform cut-off-free miRNA set enrichment analysis using miEAA [26,27] for all miRNAs instead of selecting only a subset of miRNAs. The gene ontology categories were extracted from the miEAA tool using annotations from the miRTarBase (Supplementary Table S3). A direct comparison provided strong evidence supporting the notion that miRNAs correlating with age in EVs are significantly enriched in biochemical categories compared to those in the fc fraction of plasma (Figure 3a). This finding argues further towards our initial hypothesis that EVs may specifically be loaded with noncoding RNAs that exert biological processes in remote sensing cells. To understand the nature of these processes, we compared the 16 categories that are at least three orders of magnitude more significant in EVs compared to the fc fraction to the two being at least three orders of magnitude more significant in the fc fraction compared to EVs. In the former, the strongest enrichment was found for protein heterodimerization activity, neural crest cell migration, negative regulation of inflammatory response, receptor internalization, positive regulation of neuroblast proliferation, the mitochondrial envelope, the positive regulation of DNA-templated transcription and the TORC2 complex. All categories were significant at an alpha level of 0.05 following Benjamini-Hochberg FDR adjustment in the EV fraction and none of the categories remained significant after adjustment for multiple testing in the fc fraction. As an example, we present the enrichment plots for the protein heterodimerization activity for both fractions (Figure 3b). Here, the original running sum curve for the EV fraction clearly exceeds the random background distribution while for the fc fraction random distributions reach the original one. The categories with higher significance in the fc fraction included cellular response to BMP stimulus (*p* = 6×10^−5^ vs. 0.11) and negative regulation of myotube differentiation (*p* = 6×10^−7^). Here, both categories were significant following adjustment for multiple testing for the fc fraction, in the EV fraction the negative regulation of myotube differentiation remained significant following adjustment for multiple testing. This case indeed indicates that pathways can be significant in both, the fc and EV fraction. Especially one category was highly significant in both: ‘response to hypoxia’ reached a p-value of 4.3 × 10^−5^ in the fc fraction and of 9.4 × 10^−6^ in the EV fraction (Figure 3c, Supplementary Table S3). Distinct pathways specifically enriched in the EV fraction open the question of potential effects on gene regulation in different tissues.

The core hypothesis of our work stipulates specific loading of EVs, notably considering both possibilities, in- and outside-bound, with non-coding RNAs, first and foremost miRNAs, enabling the control of specific cellular functions and gene regulation in remote cells. To identify tissues most likely affected by the EV- and fc-RNA cargo, we next combined the miRNA data generated in this study with our previously established bulk- and single-cell murine tissue-ageing atlas, *TMS* [9,10]. In these studies, we reported both linear and nonlinear ageing trajectories in gene expression signals. Like the findings on noncoding RNAs observed here, the associated genes cluster with coherent biological functions, including extracellular matrix regulation, unfolded protein binding, mitochondrial function, and inflammatory and immune responses. The expression patterns are consistent across tissues, differing only in the amplitude and age of onset. In particular, fat tissues showed early ageing signals of biochemical pathways similar to those observed in the miRNA pathway analysis described above. It was previously shown that miRNAs target genes in a pathway-specific manner [28–30]. Thus, for miRNAs associated with age in the EV- or fc-RNA in the current work, we extracted the experimentally validated target genes from miRTarBase [31] and evaluated the correlation of these target genes with age in all tissues from *TMS*. Remarkably, the analysis was limited to miRNA-gene pairs with strong evidence of functional interactions, such as from reporter assays. In this context, the expected pattern is a negative correlation of target genes with age, where miRNAs show a positive correlation with age and *vice versa*. In particular, mesenteric fat, gonadal fat, the brain, white blood cells and brown fat fulfill this expectation (Figure 3d). This result is also in line with recent parabiose-mediated rejuvenation experiments, suggesting a loss of gene expression with age that is largely mimicked by rejuvenation. Likewise, the observed tissue-independent ageing patterns matched our previous results. While fat tissues generally showed the best concordance, other tissues, such as the lung or pancreas, did not. The target gene correlation for mesenteric and gonadal fat verified the increased correlation with age for miRNAs decreasing with age and vice versa (Figure 3e and 3f). This effect was more pronounced for gonadal fat in EV-miRNAs. Translating the miRNAs and genes to a target gene network identified eight core genes: *Notch1*, *Bace1*, *Hdac4*, *Igf1*, *Eln*, *Cav2*, *Insig1*, and *Scap* (Figure 3g, Supplementary Table S4), possibly reflecting physiological relevance for both signalling networks and epigenetic processes. All but *Notch1* are experimentally validated target genes of specific members of the miR-29 family (miR-29a, -b or -c), pointing at an inherent regulatory role of this miRNA family in fat tissue. Among the gene nodes, *Nanog* could be distinguished by a positive correlation with age. This gene is targeted by mmu-miR-296-5p, which is negatively correlated with age.

Of note, the correlation presented in this analysis was indirect. The fc-RNA and EV-RNA fractions were sequenced from another cohort of mice and compared to the gene expression profiles in tissues. To test whether increased levels of these miRNAs have also been detected in gonadal fat tissue of aged mice, we used small RNA sequencing data from gonadal fat tissues from TMS [32]. Specifically, we examined identical tissue specimens that were used for sequencing the RNA expression atlas. For the microRNAs from the network (Figure 3h), we observed in most cases a mixed pattern of up- and downregulation of the targets for those miRNAs. Nevertheless, members of the miR-29 family (namely, miR-29b-3p and miR-29a-3p) showed downregulation across all target genes in gonadal fat.

Due to a limited cohort size and potential challenges in using high-throughput screening based on NGS, we performed a further validation experiment. To this end, we investigated a third independent cohort of mice and analysed the relative expression of miR-29 family members along with other dysregulated miRNAs from the network (miR-455-5p, miR-139-5p, miR-146-5p, miR-1-3p, miR-34a-5p, and miR-706) in the EV-RNA of four young and two old mice using real-time quantitative PCR (RT-qPCR). The RT-qPCR results for the miR-29 family support the sequencing data and provide evidence for an increased abundance of miR-29a/b/c-3p in EV-RNA of old mice (Figure 3i). Additionally, in the gonadal fat samples of the same mice, a – however less pronounced – upregulation was confirmed upon ageing. miR-146-5p showed a similar trend and also miR-1-3p was slightly increased in the EV-fraction and GAT tissue. In contrast, miR-455-5p was down-regulated in the EV fraction and in GAT tissue. For miR-139-5p no dysregulation was observed and for miR-34a-5p and miR-706 the dysregulation in the EV-fraction and GAT tissue did not match. The strongest effects overall occurred in the miR-29 family.

In summary, the data presented here provide a valuable resource that can be used as a starting point to study the biology of circulating noncoding RNAs in the context of ageing in general. Especially in combination with published tissue-specific gene expression atlases, these data enable the community to formulate novel hypotheses on distant cell-cell communication and affected tissues during ageing, which serves as a basis for functional studies in the future. At the same time, our analysis suggests a major role of fat in ageing processes, with EV-bound miRNAs of the miR-29 family performing important regulatory events.

## Discussion

While our study presents intriguing new insights into the correlation of EV-and fc fractions and the molecular loading of EVs with noncoding RNAs in the context of ageing, it is important to mention the limitations and how they could be addressed. First, the purification of EVs is challenging and has many pitfalls [23]. Generally, the more purification steps that are applied, the less material is left, eventually requiring a pooling of samples. We decided to achieve the maximal possible purity while still leaving sufficient material for high-throughput sequencing of small RNAs, avoiding any pooling. As stated by Schekman et al. [23], healthy scepticism concerning the possible connection between EV-miRNAs and control of gene expression in target cells should remain until functional cell culture and animal studies are conducted with EVs purified by rigorous and quantitatively documented procedures, allowing depletion of lipoproteins and other non-EV contaminants and quantification and characterization of pure EVs. Similar concerns hold for the RNA molecules detected in the EV pellets, which may include RNAs that are inside the EVs, outside the EVs, or pelleted together with EVs within larger complexes. While NTA and cryo-EM do not replace purification, they verify the presence of vesicles in the samples used (Supplementary Figure S3). A second limitation comes down to the molecular measurement and annotation of the molecules. Having reached a high sequencing depth from low input volumes, the data were mapped to the standard reference databases. Whether read molecules, e.g. mapping to piRNAs represent functional piRNAs and not fragments or reads mapping to miRNAs annotated in miRBase represent functional miRNAs, is only partially known, calling for further functional validation experiments. Another challenge is the normalization of data, which in our case relies on global normalization. Nevertheless, differences between the fc-RNA and EV-RNA fractions as well as an additional targeted validation by RT-qPCR support the general high-throughput results. Nevertheless, we acknowledge that different normalization approaches can impact the results of respective studies relying on microarrays or sequencing. Additionally, we want to highlight that we considered here only the non-coding RNA fraction from vesicles. But exosomes, known as crucial systemic signalling mediators, carry also mRNA as well as proteins. Especially when secreted by cancer cells the composition of exosomes varies depending on the disease, and these components can have an impact on the development and maintenance of the tumour microenvironment [33–35]. Applying multi-omics studies to ageing-associated vesicles is now crucial to get a comprehensive model on the action of the miRNAs reported herein and due to the complex EV micro-ecosystem.

In performing the study, we paid attention to limit confounding factors wherever possible. Because an influence of the sex on organ specific RNA signatures is known [9,10], we decided to only measure female mice in this study. Of note, the pathways comprising these sex DEGs in the previous RNA based studies largely differ from those describing ageing DEGs. We thus do not have evidence that sex differences influence the transcriptional ageing profiles from those studies. Nonetheless, the amount and cargo of EVs seems to be affected significantly by the sex overall [36–40]. In future targeted preclinical studies, the role of mouse sex on vesicle cargo type and amount should be further explored.

The primary aim of our study was to develop a resource for free circulating (fc-RNA) and extracellular vesicle-associated small RNAs (EV-RNA) in ageing. While we observe broad differences between RNA classes upon ageing, pathway and network analysis highlights a limited set of miRNAs with age-related regulatory activity. Missing miRNA target interactions (MTIs) in the regulatory core network (Figure 3e) await validation in future studies. Our results however do not only pinpoint a multifaceted ageing-factor depending on the RNA class but also on the organ. Strikingly, the results of EV-bound miRNAs suggest a major role of fat tissue in the process of ageing. The miR-29 family seems to play an essential role as a regulator of a tentative core ageing network and has been described in the context of ageing [41,42]. There is an increasing body of evidence associating fat tissue or processes therein with ageing in health and disease [43]. Age-related inhibition of adipogenesis and adipose tissue senescence leads to a decline in body fat in elderly individuals and is implicated in the development of metabolic diseases [44]. One hallmark of ageing is the so-called ‘inflammageing’, which describes a low-level chronic inflammation caused by cells taking on a senescence-associated secretory phenotype (SASP) and secreting proinflammatory molecules [45]. A recent single-cell sequencing study suggests the emergence of ‘ageing-dependent regulatory cells’ in fat tissue of mice with higher age that secrete, for example, the proinflammatory *Ccl6* as a major contributor to adipose cell senescence [46]. For some of the genes in the computed core networks, there is already evidence of a contribution to ageing or age-related diseases. The growth factor *IGF1*, for example, is a major player regulating ageing on a cellular and organism-wide level [47]. The histone deacetylase *HDAC4* has been shown to be polyubiquitylated and degraded during all types of senescence [48]; however, it seems to be overexpressed in ageing muscle [49]. Similarly, deregulation was shown for *CAV2* [50] and other genes reported in this study. The association of EV-RNAs is a likely contributor to these ageing processes and is already known from cancer research. For example, miR-29a enclosed in tumour-derived exosomes has even been shown to directly bind to intracellular toll-like receptors in immune cells, generating a prometastatic inflammatory response [51]. Moreover, overexpression of miR-29a has been shown to repress adipogenesis in humans and mice due to repression of the glucocorticoid receptor as its target gene [52]. Our data, first and foremost the pathway enrichment analysis, provides evidence that miRNAs are not randomly associate with vesicles. It is hard to distinguish whether those miRNAs have synergistic or antagonistic effects. For both mechanisms examples have been published [53–55]. Of note, miRNAs with different (seed) sequences that nonetheless regulate similar gene sets or pathways exist, pinpointing synergistic or antagonistic effects [56]. Considering the set of pathways identified in our study we assume that the miRNAs in exosomes are synergistically targeting pathways but partially also exhibit antagonistic effects. Here, a systems biology analysis together with targeted validation experiments is required.

Together with the data presented herein, it is plausible to postulate that the miR-29 family might be one of the mediators for the inhibition of adipogenesis and the induction of a proinflammatory environment in ageing fat tissue. One interesting open task for future studies is to determine the cells of origin of the EVs loaded with miR-29 and clarify the regulatory functions of the miRNA in adipocytes. This finding adds to the knowledge on the importance of extracellular vesicles and the immune system interplay in ageing and immune diseases [57], together forming the complex cellular ecosystem.

## Methods

### Animals

We initially conducted the EV-RNA and fc-RNA isolation workflow on 18 female C57BL/6N mice, 14 of which resulted in a sufficient amount of biological material. Overall, we thus generated non-coding RNA-sequencing samples from mice at the age of 2 months (*n*=3; body weight (bw): 19–20g), 6 months (*n*=4; bw: 25–29g), 8 months (*n*=3; bw: 23–26g), 12 months (*n*=2; bw: 31g) and 18 months (*n*=2; bw: 34 & 41g). The age range was selected based on our previous results on age-related tissue-specific changes in gene expression that were already evident in mice 18months old (Tabula muris senis [9,10]. Leftover gonadal fat tissue samples from the TMS study were obtained as a second cohort for RNA sequencing. To assess the size distribution of vesicles and perform EM, an independent cohort of female C57BL/6N mice was used with an age of 2 (*n*=4; bw: 19–20g) and 18 months (*n*=4; bw: 29–41g). For validation experiments using RT-qPCR, a third independent cohort of female C57BL/6N mice was used (2 months: *n*=4, bw: 19–21g; 18 months: *n*=2, bw: 27–32g). The animals – excluding the existing specimens from TMS – were housed in groups on wood chips as bedding in the conventional animal facility of the Institute for Clinical & Experimental Surgery (Saarland University, Homburg/Saar, Germany). They had free access to tap water and standard pellet food (Altromin, Lage, Germany) and were maintained under a controlled 12-h day/night cycle. This animal study was approved by the local State Office for Health and Consumer Protection and conducted in accordance with Directive 2010/63/EU and the NIH Guidelines for the Care and Use of Laboratory Animals (NIH Publication #85–23 Rev. 1985).

### Blood sampling

The mice were anesthetized by an intraperitoneal injection of ketamine (100 mg/kg bw; Ursotamin®; Serumwerke Bernburg, Bernburg, Germany) and xylazine (12 mg/kg bw; Rompun®; Bayer, Leverkusen, Germany). Subsequently, they were fixed on a heating pad in the supine position. After midline laparotomy, a maximal volume of blood (~700–1000 µL) was taken from the vena cava and transferred into plasma tubes (Sarstedt, Nümbrecht, Germany). The blood samples were then centrifuged at 20°C and 10.000 × g for 5 min to remove platelets, large vesicles, and cell debris; the resulting platelet-free plasma was stored at −80°C until further use. After blood collection, gonadal fat tissue of the mice in the third cohort was collected and snap-frozen.

### Isolation of EVs

Two hundred microlitres of mouse plasma was transferred to a 1 mL open-top thickwall polypropylene ultracentrifugation tube (Beckman-Coulter, USA) and diluted with 800 µL of phosphate-buffered saline to prevent the tube from collapsing in the ultracentrifuge vacuum. Samples were centrifuged for 2 h at 4°C at 100,000 × g using Type 50.4 Ti fixed-angle rotor (Beckmann-Coulter, USA). Supernatants were carefully removed, and the EV-containing pellets were resuspended in 20 µL of phosphate-buffered saline. Samples were stored at −80°C until further analyses. The samples were characterized according to the MISEV2018 criteria [58], and NTA and EM were performed. Limitation of material (700 µl blood/animal/sample that resulted in approximately 250 µl plasma/sample) prevented application of other analytical approaches.

### RNA extraction

EV-enriched pellets further referred to as EV fractions and EV-depleted plasma referred to as the free circulating (fc) fraction were used for RNA isolation. All samples (blood from one animal corresponding to one sample) were treated separately, and no samples were pooled. EV- and fc- total RNAs were isolated semi-automated using the miRNeasy Micro kit (Qiagen, Hilden, Germany) and Qiacube isolation robot according to the manufacturer’s recommendations with the addition of 2 µL RNase-free glycogen (20 mg/mL, Invitrogen, Carlsbad, CA, USA) to facilitate RNA precipitation. For each sample, at least two replicated sequencing results were available (12- and 18-month replicates, all other time points in triplicate). Total RNA of gonadal fat tissue was isolated using a miRNeasy Mini kit and Tissue Lyser LT according to the manufacturer´s protocol. The RNA concentrations of the EV- and fc-fractions were measured using a Qubit™ microRNA Assay Kit, and fat tissue was measured using a Nanodrop (Thermo Fisher Scientific, Waltham, MA, USA).

### High-throughput RNA sequencing

Isolated EV-RNA and fc-RNA samples were analysed by Agilent small RNA chips, and 2 ng each (EV-RNA and fc RNA) was used for Illumina-compatible library preparation using the D-Plex Small RNA Kit (Diagenode, BE). The kits employ 3´-poly A tailing and template switch-based cDNA generation using unique molecular identifier (UMI)-tagged template switch oligos. After PCR amplification involving 13 cycles, libraries were purified from TBE-PAGEs. Illumina sequencing was carried out on a HiSeq2500 platform using the High Output mode for 96 cycles. Isolated RNA from gonadal fat tissue was sequenced using the MGISeq system with the standard MPS protocol as described previously [59].

### Nanoparticle-tracking analysis

Each EV preparation and each EV-depleted sample were tested using NTA to estimate the number of particles in a sample. For that, 1 µl of plasma was diluted in 1199 µL, and 1 µL of the resuspended EV pellets was diluted in 999 µL of phosphate-buffered saline to achieve a final concentration between 20 and 120 particles/frame. Samples were then measured on NanoSight (Malvern, UK) at a camera level of 15. For each sample, three captures of 30 s were acquired. Videos were then analysed at a detection threshold of 5 using NTA 3.4 software.

### Cryo-transmission electron microscopy

Three microlitres of each EV sample was transferred to a holey carbon film-coated copper grid (Plano S147–4), blotted for 2 s, and plunged into undercooled liquid ethane at −165°C (Gatan Cryoplunge3). The grid was then transferred to a cryo-TEM sample holder (Gatan model 914) under liquid nitrogen. Low-dose bright-field images were acquired at −170°C using a JEOL JEM-2100 LaB6 Transmission Electron Microscope and a Gatan Orius SC1000 CCD camera.

### RT-Qpcr

Quantitative real-time PCR of EV-RNA and gonadal fat tissue RNA was used to validate age-related expression differences of selected miRNAs. Reverse transcription was performed using the miRCURY LNA RT Kit (Qiagen, Hilden, Germany) with 100 ng fat tissue RNA and EV-RNA equivalent to EVs from 20 µl plasma as input. qPCR was performed using the miRCURY LNA SYBR Green PCR Kit with miRCURY LNA miRNA PCR Assays specific for selected miRNAs in a 10 µl reaction volume. Uniform isolation and RT efficiency were checked using the manufacturer’s recommended spike-in controls (Uni-Sp2, 4, 5 and 6). Expression differences were calculated using the ΔΔCt method with miR-191a and let-7a (in conjunction) as endogenous controls [60].

### Computational data analysis

The sample primary processing was performed with miRMaster [61] using standard parameters. The miRNAs were mapped using Bowtie (version 1.2.3) and allowing up to 1 mismatch. As reference databases we used miRbase (version 22.1); Ensembl ncRNA (version 100), RNACentral piRNA (version 15), GtRNAdb (version 18.1), NCBI RefSeq (bacteria & viruses; version 74) and NONCODE (version 5). As output, miRMaster generated a list with the expression of 80,668 RNAs from 10 RNA classes. The data were normalized to expression in one million reads and further processed with R (R 4.0.4 GUI 1.74 Catalina build (7936)). For quality control aspects, we compared the correlation of replicated samples to the correlation obtained for samples that were not replicated. In the first case, we reached an average correlation of 0.93 for the replicates and 0.80 between samples that were not replicates of each other (*p* < 10^−10^). Venn diagrams were generated using the *eulerr* package from R. Mapping the fraction of variance to different parameters was performed using the principal variance component analysis (*pvca*) package. Splines were computed using the *smooth.spline* function with seven degrees of freedom. Colour palettes were generated using the *RColorBrewer* package. Smoothed scatter plots were computed using the *smoothScatter* function setting the point number to 500. Clustering was performed for the most highly expressed noncoding RNAs (at least 5 reads per million in at least one sample) using the scaled expression matrix (z-score of each feature). The clustering was performed with the *hclust* function using the Euclidean distance measure. Clusters were extracted by cutting the dendrograms at 1/1.25 of the maximal height. Heatmaps of target genes were computed using the *heatmap.2* function. Network visualization was performed using *iGraph*. As input for the network analysis, targets from miRTarBase [31] were used; however, they were restricted to strong evidence targets (i.e. experimentally validated). To compute the statistical concordance of RNAs correlated with ageing across sample types, a random background distribution with respect to positive and negative correlation was assumed. Briefly, a random distribution would mean that close to 25% of non-coding RNAs is consistently positively regulated in plasma and EVs, 25% is consistently negatively correlated with age and 25% in each are positively correlated in the one and negatively correlated in the other specimen type. Where applicable, p-values were corrected for multiple testing using the Benjamini Hochberg method with an alpha-level set to 5%.

### Pathway analysis

For the pathway analysis we used miEAA 2.0. Precisely we performed a miRNA set enrichment analysis of the mature RNAs. To this end, we sorted the correlation value of the EV- and fc-fraction separately with the age and uploaded both sorted lists to miEAA. As organism we selected *mus musculus* and choose the gene ontology categories derived over the miRTarBase. We then selected a p-value threshold of 1 to force miEAA reporting of all nominal p-values, facilitating a direct comparison between the pathway results of the fc- and EV-fraction. We adjusted the p-values using the Benjamini-Hochberg method. As graphical output we present the enrichment plots. These plots describe the enrichment statistics for the input list as solid blue line. In the background, the same distributions computed for random lists (corresponding to the result of non-parametric permutation tests) are shown.

### Matrix factorization

We predicted the age of samples with respect to three age groups: ‘young’ (2 months), ‘middle’ (6–8 months) and ‘old’ (12–18 months). To this end, the expression patterns were split into 20 individual matrices for each of the 10 noncoding RNA classes and for plasma and EVs. We first normalized the given nonnegative Matrix D by dividing all elements by the maximum value in D.To obtain the probabilistic regarding the age groups, we decomposed the matrix D into two further matrices T and P, where P gives us the desired probabilities. Tstands for the matrix of the typical age group vectors, i.e. in each entry of a column, there is a value representing all entries at this position of all samples belonging to this age group. The matrix P contains the probabilities of each sample to each age group respective to their typical vector in T. We formulated the non-negative matrix factorization as the optimization problem:

$$\begin{array}{c} Given:D\in R^{m\times n}\\ Unknown:T\in R^{m\times 3},P\in R^{3\times n}\\ Solution:\underset{T,P}{\min}|\left|D-TP\right|{|}_{F}^{2}\\ s.t.0\leq T\left[i,s\right]\leq 1\forall i\in \left\{1,\ldots ,m\right\},\forall s\in \left\{1,2,3\right\}\\ 0\leq P\left[s,j\right]\leq 1\forall s\in \left\{1,2,3\right\},\forall j\in \left\{1,\ldots ,n\right\}\\ \sum {\text{\textbackslash{}NOlimits}}_{s=1}^{k}P\left[s,j\right]=1\forall j\in \left\{1,\ldots ,n\right\} \end{array}$$

The first two constraints require all entries of the matrices T and P to lie between 0 and 1. Since we were interested in the percentage of a sample belonging to the three age groups, we also required all columns of the matrix P to sum up to 1 using a numerical solver [62].

We then classified each sample by choosing the index with the highest entry of each column in P and assigned the index as a label to each one. However, the rows of P were invariant to permutations. Here, this means that it is not clear which label corresponds to which age group. Furthermore, a measure of quality for the results was needed. Using the known age, we could construct a ground truth for each sample and calculated the classification accuracy for every permutation. The ground truth used was ‘young’ corresponding to two-month-old mice, ‘middle’ to six- and eight-month-old mice and ‘old’ to twelve- and 18-month-old mice. Finally, we chose the permutation labels that maximize the accuracy and obtain a measure of quality.

## Supplementary Material

Supplemental Material

## Data Availability

All sequencing data are freely available from the Gene Expression Omnibus under accession number GSE222857. All raw fastq files can be downloaded from the corresponding sequence read archive (SRA) entry. https://www.ncbi.nlm.nih.gov/geo/query/acc.cgi?acc=GSE222857

## Code availability

The primary data analysis was performed using miRMaster. The software is available as a web service. The other analyses were carried out using standard R packages that are freely available (see Methods Section).
